# Round hole, square peg: a discourse analysis of social inequalities and the political legitimization of health technology in Norway

**DOI:** 10.1186/s12889-019-8023-3

**Published:** 2019-12-16

**Authors:** Daniel Weiss

**Affiliations:** 1 HUNT research center, Department of Public Health and Nursing, Norwegian University of Science and Technology, Levanger, Norway; 2 CHAIN research center, Department of Sociology and Political Science, Norwegian University of Science and Technology, Building 10, Dragvoll, 7491 Trondheim, Norway

**Keywords:** Social inequality, Health, Technology, Innovation, Discourse analysis, Norway

## Abstract

**Background:**

As research increasingly investigates the impacts of technological innovations in health on social inequalities, political discourse often promotes development and adoption, limiting an understanding of unintended consequences. This study aimed to investigate national public health policy discourse focusing on innovative health technology and social inequalities, from a Norwegian context.

**Methods:**

The analysis relies on a perspective inspired by critical discourse analysis using central State documents typically influential in the lawmaking procedure.

**Results:**

The results and discussion focus on three major discourse strands: 1) ‘technologies discourse’ (types of technologies), 2) ‘responsibility discourse’ (who has responsibility for health and technology), 3) ‘legitimization discourse’ (how technologies are legitimized).

**Conclusions:**

Results suggest that despite an overt political imperative for reducing social inequalities, the Norwegian national discourse gives little attention to the potential for these innovations to unintentionally (re) produce social inequalities. Instead, it is characterized by neoliberal undertones, individualizing and commercializing public health and promoting pro-innovation ideology.

## Background

### Introduction

We have long understood the powerful potential of innovative technologies when developed and adopted by society’s individuals and institutions. These resources can afford often inconceivable benefits and are frequently necessary to elevate or sustain positions in the prevailing social or political hierarchy. In contrast, the decision to ignore or abandon the development, adoption or implementation of innovative technologies is also often a decision to relinquish social, economic, cultural, or military superiority and power.

Political discourse and arguments of social progress and superiority have contributed to a persistent and widespread positive bias for the development and adoption of technological innovations in society. This attitude has dominated the public policy sector, where there has been a push to reform *innovation* from a historically negatively loaded term to a positive one [1]. As a result, innovation, particularly in relation to technology, has largely become an undisputed practice [1–3]. This perspective is naturally spilling over to policies related to health technology, where the often argued ‘neutrality’ of technological innovation can be questioned [3, 4].

Although technological innovations have proven, in many cases, to be extremely useful and effective, uncritical development and adoption of these technologies opens for a myriad of unanticipated and often undesirable consequences that undermines their aggregate social value. Recent research has begun to investigate and document these effects, presenting a complex picture of health technologies [2, 3, 5–8]. These consequences are increasingly being recognized as mechanisms that have the potential to (re) produce social inequalities in health [4, 9–12]. It is increasingly clear that technological innovations produce winners and losers, as is apparent with innovative technologies entering the labor market but also those entering the health sector. Attention for these associations comes at a time when social and political awareness is increasing for understanding the mechanisms that are leading to a modern growth in national and international social inequalities, including those in health [13–17].

Therefore, understanding how current political ideologies are addressing the association of an increasingly technology-affected health sector with growing inequalities in health becomes relevant for understanding the social value and broad impact of these innovations [8]. Analyzing political discourse has been highlighted as an effective method of exposing political ideology while also investigating the ways through which official use of text (re) produce dominance, social hierarchies and inequalities [18–20].

### Norwegian context

The Norwegian case is interesting for a number of reasons. Firstly, Norwegian public health policy is bound by law to promote health and reduce social inequalities. The Norwegian Public Health law of 2012 explicitly states, ‘The goal of this law is to contribute to a social development that promotes public health, including reducing social health inequalities’ [21]. Secondly, the Norwegian commitment to be an international leader in the development, adoption and implementation of health technologies is significant, structurally integrating this work into government agencies and policies. The Norwegian government, as of 2016, has a Directorate for e-Health with a wider mandate for organizing, implementing and guiding policies and technologies in e-Health and information and communication technologies (ICT) in the health and care sector. This directorate is a product of almost 30 years of political commitment in this area, starting with the creation of the Norwegian Competence center for information technologies in the health and social sector (KITH) in 1990. These efforts have in part contributed to high overall rates of household internet access across income categories (93% for household income between 0 and 299,000 Norwegian kroner compared to 99% for households with an income over 900,000 Norwegian kroner) [22]. However, age and gender inequalities persist in the use of internet-based technologies, where, for example, over 66% of men age 16–34 years have used the internet to search for health-related information during the previous 3 months compared to under 40% for those aged 65–79. For women this gap is even larger, with over 86% between the ages of 16–34 and only 51% between the ages of 65–79 [23]. Furthermore, some research has suggested that there is large variance in the types of users of internet-based activities and that age and gender often determine significant variations in use patterns [24]. Furthermore, non-users generally have lower levels of education and are often unemployed [25]. Although these numbers far from fully represent the complexity of socially constructed inequalities and digital technologies, one could argue that focusing merely on remedying these inequalities is unjustified as they may be shaped by differing views on the use, and importance, of the internet and digital tools in society by various social groups that do not necessarily correspond with a single standard of social conformity. These inequalities, however, (particularly when coupled with access to broad social benefits being dependent on digital or internet-based tools) may contribute to reproducing other fundamental, and inherently unfair, social inequalities, such as inequalities in health. In this regard, Norway has repeatedly been referenced in relation to an elusive ‘Nordic paradox’, where one would expect low levels of inequalities in health due to generous welfare policies and a focus on promoting social equality, but where these inequalities instead remain relatively large and persistent [16, 26, 27].

### Interest and aims

Based on the information above, the broad interest of this study is to investigate the national public health policy discourse with specific focus on innovative health technology and social inequalities. More specifically, the central questions under investigation in this study are the following:

1. How is responsibility in the discourse assigned for the development, adoption, and implementation of innovative technologies in health and how is this discourse entangled with a more general discourse of responsibility for health?
2. How are innovative health technologies legitimized in the discourse and how is this discourse entangled with a social inequalities in health discourse?

## Methods

### Documents

The documents in this study focus solely on political discourse (i.e. a single discourse plane). The analysis includes a total of 33 central strategic planning documents – such as white papers – and government reports from various government departments and agencies as well as specific plan and strategy documents from the Norwegian Directorate for e-Health (see Appendix 1). All these documents are typically influential in the lawmaking procedure [19, 28]. Included documents addressed health technology in a public health policy context.

### Data collection

Similar to methods of grounded theory, the systematic collection of documents analyzed in this study was not a distinct, isolated stage but rather a continuous and recurring process throughout the study period [19]. Recommendations for central documents by way of consultation with representatives from the Norwegian Health Directorate and the Norwegian Directorate for e-Health were followed by a hand search of government websites and historical archives. Following the collection of a sample of central documents, a snowball method was used to collect additional documents of relevance that were referenced in the originals (see Appendix 1).

Only documents after 1997 were included in the analysis as this date has been specifically referenced in several documents as the year in which an official government agenda surrounding health technologies began [29, 30]. Some documents were not analyzed and coded in their entirety as some broad public health documents included large sections of content irrelevant for the aims of this study (i.e. unrelated to *both* health and technology; see Appendix 1 for more details).

### Analysis

The analysis relies on a perspective grounded in a critical research approach to analyzing political discourse, an area relevant for public health concerns [31]. Critical research is often characterized by a focus on social inequalities, power relations, politics, and issues related to agency and empowerment that “shifts research away from the production of knowledge for knowledge’s sake and edges or nudges it towards a more transformative vision of social justice” [32, 33]. This analysis is therefore inspired by various methods of Critical Discourse Analysis (CDA), a well-developed field of study also focused on research methods that critically analyze how hegemony and inequality are (re) produced and legitimized in text and talk [18, 19, 28]. Although a single, distinct set of methodological criteria for CDA are rarely referenced (and in fact typically resisted in the literature), this study follows a structure grounded in Jäger and Maier’s methodological outline [19]. Where the methodological approach in this study departs from traditional CDA is the absence of detailed linguistic operationalization of discourse fragments, often focused on analyzing structural aspects of the text at the level of the sentence or word, such as lexical style, word order, and syntactic and propositional structures. Instead, this study focuses on a thematic analysis of the material, however drawing inspiration from CDA’s theoretical and methodological approach to critically identifying contradictions in dominant strands of discourse that may reproduce or legitimize existing inequalities in power. By drawing inspiration from CDA, and applying Jäger and Maier’s general structural analysis of discourse strands, this analysis has been able to identify and disentangle broad dominant strands of political discourse that represent institutionalized forms of, what Bourdieu would refer to as, symbolic violence (namely, the misrepresentation of unconscious reinforcement of existing imbalances in power in society as legitimate forms of social normalization) [19, 34].

All coding and analysis were performed using Nvivo. Coding focused on three major themes: 1) reference to specific technologies and their technical definitions; 2) responsibility for technology adoption and diffusion in a health context; and 3) general attitudes towards technology, with an ancillary perspective grounded in social inequalities. The material included in this analysis (see Appendix 1 for more details) was first coded using a broad range of codes representing four general thematic areas of interests: 1) Understanding of technology and innovation, 2) Understanding of public health and social stratification, 3) Reference to action and policy, and 4) General (see Appendix 2 for a full list of codes used in the analysis). The identification of relevant codes followed a combination of inductive and deductive processes, where some central codes were identified a priori, in part in relation to the findings from a systematic review on a similar topic [9]. Additional codes were then identified during initial examination of central documents first collected on recommendation by relevant authorities. Throughout the snowball process, additional codes were identified and/or incorporated into existing codes if new documents presented relevant information that was not present in previous documents, but which added to new perspectives relevant for the aims of this analysis or one or more of the thematic areas of interest. In addition, summary notes were written for each document. Following this initial coding, all coded material was recoded with a focus on organizing the material into the following discourse strands: types of technologies dominating the discourse (resulted in subcodes ‘biotech’, ‘e-health and IT’, ‘electronic journals’, ‘welfare technologies’, and ‘others’); the assignment of responsibility at various societal levels (resulting in subcodes ‘health care institutions’, ‘multisectoral, general’, ‘private, general’, ‘private, individual’, ‘private, industry’, ‘public, general’, ‘state, national’, ‘state, regional’, ‘state, local’); and, attitudes towards technology, or political ideology as it relates to health technology (resulting in subcodes ‘pro-innovation’, ‘legitimization, commercialization’, ‘legitimization, empowerment’). The subcodes represent the emergence of dominant themes within each discourse strand. A conceptual mapping exercise was used in order to further reduce the quantity of data (using both document notes and the remaining coded material), identify the importance and weight of existing discursive themes, identify and organize discursive entanglements, and organize dominant themes diachronically (Appendix 3).

## Results

### Technology types & definitions: themes surrounding the discussion of specific technologies

Discussions of technologies are dominated by a focus on e-Health and ICT technologies, electronic patient journals, welfare technologies, and biotechnologies. Although there is often significant overlap between these groupings, the technologies within each of these categories are often discussed in isolation, with distinct definitions and objectives.

### E-health & ICT

E-health technologies dominate in the early years (1997–2005). During these years, there is a heavy focus on the use of the internet (more generally as a platform for sharing information using online portals, databases and websites) and internet-based communication technologies (email, telemedicine, online booking). Tools to access and use the internet (mobile phones, computers, tablets) are naturally a large part of this discourse from early on but it is not until after 2010 that we begin to see technologies like modern applications for smart phones and tablets enter the discourse (m-Health), strengthening in more recent years. The years after 2010 also mark the rise of monitoring and surveillance technologies (portable, wearable or home-based sensors and measurement devices). The post-2013 years also see big data, cloud-computing, robotics, and the internet of things enter the general discourse. Throughout the study period, e-Health technologies are discussed in relation to health services settings, however discourse centered around technologies become increasingly focused on consumer, (digital) self-service, and home-based technologies, particularly in the years after 2013.

### Electronic patient journals

Although electronic patient journals (EPJ’s) are themselves an e-Health technology, they are often prioritized as a distinct technological innovation. EPJ’s begin permeating the general discourse in the years following 2000. They quickly become a central and persistent thread. Their dominant position is strengthened in the years following 2008, with a focus on implementing a streamlined national EPJ system (called *‘one inhabitant – one journal’,* outlined in the white paper with the same name in 2012). EPJ development is, throughout the study period, discussed in conjunction with, and dependent on, an internet-based platform used to offer various individualized services.

### Welfare technology

Welfare technologies, as a distinct group of technologies, enter the general discourse in the years following 2010 (however some of the individual technologies later classified as welfare technologies appear in the discourse before this). Not coincidentally, this dominant position in the discourse coincides with the term ‘welfare technology’ being more concretely defined and its use becoming more universally recognized (politically, technically, etc.) around the year 2010/11. Although we see a strong overlap with e-Health, welfare technology after 2010 is often addressed as a distinct technological innovation, divided into four categories: 1) Safety and security technologies (such as alarm systems that monitor various conditions of the individual or the home); 2) ‘Compensation and wellness’ technologies (technologies that compensate for reduced physical or mental functioning such as robotics, smart home technologies, home-based physical activity and rehabilitation technologies, and automatic scheduling technologies such as electronic medication reminders); 3) Technologies for social contact (such as video communication, social media, the internet, and robotics); 4) Treatment technologies (such as patient journals, technologies for information and communication sharing with health personnel, and sensor technologies that monitor, record and send health-related information). Discussions of specific technologies are inspired largely by technologies that act as logistical aids, sensors for 24-h surveillance and monitoring of both the home and the patient/individual (with GPS capability for example), remote home-based communication, and home-based treatment, analysis and care.

### Biotechnologies

Although biotechnologies are mentioned in documents before 2010 (gene technologies, systems biology, designer medications, and biological implants such as sensors and micro/nanotechnologies), biotechnology does not become a dominant part of the general discourse until the years following 2010. Focus is given to molecular and gene-based technologies (gene sequencing and testing, diagnostics and therapies) and novel prescription medications (including advanced therapy medicinal products), sometimes mentioning stem cell, biological implant and nanotechnologies. The gene-based technologies also inspire discussion of the value of personalized medicine as an innovation (witnessed in part in a 2011 document detailing the national strategy for biotechnology).

### Other technologies

Other technologies enter the general discourse from time to time but tend to be much less influential when compared to those listed above. Technologies of note, however, include innovations to more traditionally institutionalized diagnostic and treatment technologies (such as mobile x-ray and ultrasound devices, MR, CT, PET, image guided surgery).

### Technology, health and the ‘responsibility discourse’

Disentangling discourse strands relevant to the assigning of responsibility for public health (broadly defined) and responsibility for health technologies (from development to adoption) resulted in the emergence of the following trends in what we are calling the ‘responsibility discourse:’ 1) consistent general State oversight and promotion; 2) a transferring of increased responsibility to the local level; and 3) a continued focus on strengthening public/private partnerships.

### The state

While the State assumes central responsibility for ensuring equal services and population-wide public health throughout the study period, the responsibility discourse is increasingly framed within the confines of empowering the individual. Here, focus is on the State’s responsibility to ensure equal opportunity while challenging the individual to assume greater responsibility for personal health.

‘It is about finding the right balance between the individual’s responsibility for one’s own life and the authorities’ responsibility for creating the most equal conditions possible.’ [35]

For health technologies, the State assumes responsibility for setting national standards as well as coordinating and constructing a national infrastructure for implementation, particularly for e-health/ICT. The State accomplishes this through its departments, directorates and organizations for research and innovation. After 2012, focus increased on the State’s role as a major purchaser of health technologies and an agent for pro-innovation regulation.

‘The Government has an objective of increasing the degree of innovation in the health, care and welfare services, and for the public sector to be a driving force for, and active user of innovation.’ [36]

This is to be accomplished primarily through a national center for health-IT and welfare technologies – for which the national Health Directorate is assigned increased responsibility. In 2016 the e-Health Directorate is established and given responsibility for strengthening the State’s role in e-Health management, financing, delivery and organization at the national level. Similarly, a national center for e-Health research is created to ‘collect, produce and disseminate knowledge needed by authorities to develop a knowledge-based e-Health policy’ [37]. This primarily to increase the pace of development and implementation of technology in this sector.

‘National governance … management, financing, delivery, organization and implementation of e-Health shall contribute to realizing e-Health in a faster and more cost-effective manner.’ [38]

### Local level actors

From the mid-2000’s a general focus on transferring responsibility to the municipality level intensifies. This transfer of responsibility to local state actors is further strengthened with a legal precedence anchored in a documented national coordination reform for public health [39] released in 2009 and which went into effect in 2012 (the same year as the new national public health law).

‘ … the projected growth in needs within a collective health service must as far as possible find solutions in the municipalities.’ [39]

‘The municipalities themselves have responsibility to exploit opportunities that lie in new technology … ’ [30]

Although the State continued to assume responsibility for national coordination, municipalities are increasingly expected to assume responsibility for making local-level decisions concerning the implementation of public health services and the availability of health technologies. It is argued that through decentralized decision-making at the municipality level, health promotion and prevention efforts can be more effective, and available technologies can more effectively meet local needs. This includes municipalities strengthening their role as the State’s purchaser of health technologies and promoter of public sector innovation, but also increasing private sector business development at the local level.

‘The municipalities have a central role in public health work across different sectors, in primary services and in business development.’ [40]

Focus on private individuals also increases, particularly as interest in transferring responsibility to the local level intensifies. From the beginning of the study period, the discourse in general stresses the importance of individual choice and responsibility, but continues to mention the importance of structural and systemic environmental characteristics.

‘Individuals and communities have responsibility for public health work, but the population’s health is not least a result of developments and political choices beyond the reach of the individual.’ [41]

A responsibility discourse focused on individuals strengthens throughout the study period, with the emergence of an ‘empowerment discourse’ gaining strength in the mid 2000’s, complimenting the ‘responsibility discourse’ and focusing attention on increased user involvement. Discussions of user involvement center on a transfer of greater freedom and control to the individual, improving service delivery and more effectively meeting the needs of the user. A further, detailed explanation of who these users are is however missing from the discourse.

In the wake of the 2009 national coordination reform, this empowerment discourse again strengthens into an *expectation* of user involvement in both the delivery of health services and the formation of public health efforts, but also in the adoption of health technologies. Although user involvement is presented as a means of empowering the individual, the empowerment discourse also provides legitimacy for the transfer of an increased amount of responsibility to private individuals.

‘Measures for improving patients’ and users’ ability to care for their own health contributes to a better quality of life for the individual, and to the development of a more sustainable health and care service … It is also crucial that patients and users are encouraged to set their own goals for health and health behavior, and are not just passive recipients of others' advice and recommendations.’ [42]

‘Although the public sector accounts for much of the health and care sector procurement, we expect users and their relatives to become an increasingly important customer group that will demand technology, such as tablets and digital measuring devices.’ [43]

As these ‘responsibility’ and ‘empowerment’ discourses evolve, health technologies are themselves increasingly seen as an active resource for supporting and promoting the effective transfer of responsibility.

‘Technology will challenge people to take responsibility, both for welfare programs, their own life and in relationships to other people in daily life.’ [44]

‘New technology gives patients more responsibility and control.’ [45]

Home-based health technologies are seen as central to this objective. These technologies provide an opportunity for physically relocating the point of services, and therefore responsibility, to settings controlled by individuals and, to a lesser extent, municipalities. The empowerment discourse contributes to emphasizing the importance of innovative health technology and further legitimizing its development, adoption and use.

‘The monitoring of one’s own health, home-based solutions and technology that can help people remain at home for as long as possible, will be important with respect to sustainable development, disease prevention, improved quality of life and active ageing.’ [36]

### Public-private partnerships

Focus on strengthening public-private partnerships, by investing in innovative health technologies, for delivering health services and general public health is central to the responsibility discourse throughout the entire study period.

‘The Norwegian health and care sector needs an improved interaction with the business sector to achieve its goals.’ [40]

Public-private partnerships are justified as a means of improving health services throughout the sector, but are also presented as a means of commercializing these technologies, by strengthening and supporting a health technologies industry, and therefore promoting national value creation. The research community is presented as a central State agent for strengthening this partnership, by using public funds and research grants to support private sector technology development and transfer.

"Today the industry is small, but it can become a growth industry with global potential... A business community with strong and innovative companies that embraces innovations from the research community is a prerequisite for good health and welfare services in the future.’ [40]

Municipalities are again challenged to take increased responsibility for health technologies by partnering with industry to develop and implement effective technologies to innovate and streamline service delivery (i.e. an ‘innovative public purchaser’). Local and regional healthcare institutions are expected to be actively involved in these efforts, to test and implement technologies. Municipalities are also expected to involve individual users in development and implementation processes. Focus on involving these partners, particularly individual users, once again connects the responsibility discourse to the empowerment discourse, with a stated goal of better integrating user needs. However, a discussion around whether strengthening public private partnerships is an effective political strategy for achieving this goal seems mostly assumed and expected.

‘Municipalities have also had close cooperation with suppliers to improve products so that they better meet user and service needs.’ [46]

‘The public sector constitutes an important domestic market for Norwegian industry. Purchasing through innovative acquisitions … is an important tool.’ [40]

Focus on building and strengthening public-private partnerships intensifies in the post-2013 years.

### Technology, health and the ‘legitimization discourse’

Throughout the study period, the discourse is highly partial to positively representing health technologies. Although some of the challenges associated with these technologies are at times discussed in detail, focuses tends to be on technical and security issues, which are seen as barriers to the development and implementation of these technologies. The technologies themselves are rarely questioned and broader social concerns are largely ignored. Although questions of social inequality are sometimes referenced, attention is mostly on regional inequalities, based on variations in municipal priority-setting and financial resources. Issues of social inequalities are rarely addressed, and technologies are often seen as likely of reducing social inequalities as they are a mechanism for increasing them. This positive representation of health technologies leads to a discourse increasingly focused on legitimizing the role of health technologies (i.e. the legitimization discourse).

### Pro-innovation (technology) bias

A pro-innovation bias dominates throughout. Technologies are presented as a necessary resource for the proper functioning and effectiveness of health and welfare services. Promoting the adoption and diffusion of these technologies is therefore explicit in the discourse.

‘eHealth is the single-most important revolution in healthcare since the advent of modern medicines, vaccines, or even public health measures like sanitation and clean water.’ [30]

‘Medical technology, welfare technology and new innovative solutions must be developed and implemented.’ [47]

This legitimization discourse tends to emphasize the pressing nature of rapidly promoting adoption and diffusion of these innovations and stress the inevitability of a technology-based public health service. Furthermore, rather than discussing broader potential social consequences of these innovations, the consequences of *not* adopting are often insinuated to strengthen the power of a pro-innovation and pro-technology ideology.

‘We are facing a rapid development in medical technology and welfare technology.’ [47]

‘It is necessary to focus on innovation, knowledge and technology in order to meet the challenges in the sector, as well as to facilitate safe, high-quality services, renewal and industrial development.’ [36]

This pro-innovation ideology continuously emphasizes the benefits of these technologies. These benefits tend to be grounded in prevailing social values, such as government efficiency, individual freedom, quality and safety, and economic growth, adding strength to this pro-innovation ideology. Whether these benefits are based on reliable and representative data for specific technologies or a general faith in innovative technologies is sometimes unclear.

"Demands for action, belief in progress and expectations of increased prosperity and welfare are among the main driving forces behind the demand for new technology." [48]

### Legitimizing health technology

Discourse strands focused on public empowerment and market potential are used to further legitimize the development, adoption and implementation of health technologies, defending a general pro-innovation ideology.

As a focus on increased user involvement and responsibility evolves, so too does the empowering capabilities of innovative health technologies – connecting the legitimization and empowerment discourses. Health technologies are presented as effective tools for promoting empowering social processes such as democratic decision-making, the personalization of services, and an increase in individual freedom, control and autonomy. The legitimization discourse however is ambiguous in discussing whether these technologies have in fact demonstrated these effects or whether these effects are simply expected and desired. Additionally, whether unanticipated and undesirable consequences could potentially undermine or outweigh the positive capabilities of these technologies is left completely unaddressed. In general, it is assumed that the empowering effect of these technologies will consequently improve quality of life for adopters and users.

‘Increased use of welfare technology will give new generations of older people and other user groups more choice, increased security and independence and greater opportunities for participation in social life.’ [44]

‘The use of technological facilities for localization, such as the use of GPS, can help to provide greater freedom for patients/users in that they can go out without a follower, which will be important and increase the quality of life for many.’ [49]

The legitimization discourse leans on a general assumption that the public desires and demands technological innovation and is generally familiar with and satisfied with the general development and direction of health technologies in society. These statements however rarely contain reference to information that may in fact support these claims.

‘At the same time, users, patients and society have expectations that ICT in the healthcare system will develop in line with the development they know from other areas of society.’ [50]

Furthermore, a general presupposition that technological innovation will invariably create value in society is persistently used to legitimize the development, adoption and diffusion of technological innovations. The research community is expected to be an active stakeholder in these efforts, explicitly contributing to the development of products, resources and research results that can be patented and commercialized. By the late 2000’s the market potential of technological innovations in the health sector is strongly embedded throughout the general discourse. The ability to innovate is explicitly linked to an ability to create value. There is a general representation that innovation is, and always has been, the foundation of the welfare state.

‘Innovation has always been a central source of value creation and for the development of the welfare society.’ [51]

‘The Government will support the development of health-friendly business as a political priority area for innovation and industry.’ [52]

The State’s role is therefore to support private sector innovation with the justification that innovative health technologies are a mechanism for driving both large national economic returns as well as improving public health services more generally. Innovation, particularly technological innovation, is presented as nothing other than a win-win for all sectors of society.

‘The health industry can be described as an industry with double gains. The advances that are made contribute to welfare and health while simultaneously creating value and jobs.’ [45]

Whether this is truly the case is rarely investigated, or in any case presented, in a comprehensive way. Moreover, the legitimization discourse suggests a dominant ideological positioning of innovation, particularly technological innovation, as a means of promoting a particularly economic international competitive advantage.

‘Stronger industrial development in the health and care sector … will also ensure improved conditions for the Norwegian private sector in terms of technology development and service innovation in a broad and growing global market.’ [36]

The technological innovation paradigm therefore becomes a political ‘necessity’ that must be exploited to a much larger degree. Attention is given to the significance of negative economic (as well as social) consequences of slow or no technological innovation, while simultaneously highlighting the endless benefits of increased innovation. After 2013, particular attention is given to internationalization and the development of an export market for these innovations.

## Discussion

Throughout the study period, a number of trends emerge. E-health and welfare technologies dominate as broad (but sometimes overlapping) categories of prioritized health technologies, with specific focus on innovative technologies that improve capabilities for monitoring, surveillance and self-care. Responsibility for applied development, adoption and diffusion of these technologies is dominated by a focus on the role of municipalities and individual users. The State however retains ultimate control over the general positioning of these technologies, with a growing interest in forming partnerships with, and supporting, the private sector. Moreover, the innovative potential of these technologies is presented as socioeconomically positive and efforts to legitimize these technologies focus on individual empowerment and the promotion of national wealth and economic competitiveness.

It is, however, important to note that the discourse reflected in the results and discussion of this study, while a dominant one, is one of many discourses. This study is designed to investigate political and ideological discourse that has the potential to reinforce mechanisms of social dominance and hierarchy. It is therefore a perspective investigating the dominant political discourse surrounding technologies in health while grounded in a contextual focus on socioeconomic inequalities. Other perspectives would, of course, highlight a myriad of other discourses that exist alongside this discourse.

### Legitimization – goal or consequence

It may appear that the overall legitimization of innovative health technologies is driven by an explicit and ideological aspiration to actively support and promote the development, adoption and diffusion of these innovations. However, this legitimization discourse may rather be a consequence of the influential power of innovative technologies in society (i.e. non-neutrality). Under current economic incentives, innovative technology development is both inevitable and imperative. The traditional economic rationale is one where social welfare is a product of economic growth, where economic growth relies on corporate advantage, and where corporate advantage is encouraged by innovative product development [1]. Technology has therefore become synonymous with innovation and innovation synonymous with economic superiority (i.e. ‘innovate or die’), echoing sentiments of technological determinism [53, 54].

When innovative technologies underpin national identities and economic superiority in a globalized economy, it becomes imperative that these technologies be politically institutionalized in order to gain control over them [1]. It can then be argued that legitimization is a natural consequence, rather than a goal, and therefore becomes a central theme in political discourse. Public health and care services become just another sector in society to be affected, as technological innovations expand into this sector and the promise of commercialization and economic efficiency grows [2, 3].

From the perspective of the present discourse, this legitimization presents itself as a well-known semantic strategy characterized by positive self-representation and negative other representation [18]. In this case, the pro-innovation bias dominating the present discourse (as well as much of modern Western political discourse), represents technological innovation (the ‘desired self’) as inherently good for society and simultaneously represents the non-technological alternative (the ‘other’) as negative or counterproductive to society’s values and desires [1]. Innovative technologies are rarely problematized and, when are, this is most often in relation to barriers that impede on political aspirations such as safe and socially acceptable implementation of these technologies. Larger social concerns that may question the position or aggregate value of technological innovations in society are left largely unaddressed. This discussion is instead replaced with a pro-innovation ideology and a legitimization discourse (what some also characterize as a neo-liberal discourse) focused on empowerment, self-responsibility, and economic advantage [2, 3, 5].

This strategy of positively self-representing innovative health technologies, and negatively representing any alternative, is a way of managing the impressions of technological innovations [18]. Managing impressions of technological innovation in the political discourse provides the government with a method of gaining control over technology’s position in society. This control allows for directing socioeconomic priorities and general social acceptance of technological innovations, while strategically positioning the government to capture (often economic) benefits that might accrue from these technologies, even if many of the social benefits that tend to dominate the political discourse lack sufficient scientific evidence [7].

### Social inequality – an unintentional and undesirable consequence

The discursive legitimization of innovative health technologies with a focus on empowerment begs the question: Who is empowered by these new technologies? The emerging trends in these documents provide a clear indication that the government will actively promote the development, adoption and diffusion of new technologies in health, particularly through national policy-making and municipal responsibility. Simultaneously, the government has a clear agenda to provide equal health and care services for all, which the government is in fact bound to by law following the introduction of the 2012 health law. However, the discourse surrounding health technologies represented in these documents presents several relevant paradoxes that are left, at best, unaddressed and, at worst, unrecognized.

First, these apparently empowering technologies may not result in an aggregate increase in independence but instead merely relocate the source of dependence. Technologies that dominate the present discourse are those that geographically relocate service and care in and around the home and body of the patient or user. This is presented as a means of freeing the individual from using traditional, institutionalized services and empowering (or challenging) users to gain increased control over their own health and activities of daily living. However, this independence from traditional, institutionalized services also increases daily dependence on technological aids, in some cases using the empowerment discourse as a justification for creating entirely new technologically dependent interventions, including replacing activities not traditionally delivered by the health sector [6, 7]. This type of ‘personalized,’ rather than ‘institutionalized,’ dependence on technological aids also has the potential to increase individual dependence on the, often commercial, producers of these technologies and the consequences this dependence may promote, as Lupton has also highlighted and discussed in detail [2, 55].

Relevant for social inequalities is the potential that a loss in autonomy resulting from these technologies is strongest for low socioeconomic status (SES) individuals, who are often less active and engaged users and therefore capture fewer benefits [10]. Conversely, these technologies may increase autonomy for higher SES individuals who often experience better overall health, are more active and engaged users of these technologies, and have a number of other resources at their disposal to promote or improve health [9, 11, 14]*.* This may result in what can be characterized as a technology-based double burden for low SES individuals, who generally obtain less overall benefits from these technologies yet are more dependent on the benefits they manage to obtain.

Secondly, as suggested by the previous argument, a resource such as innovative technologies, with genuine potential to offer benefits to society, has an equally strong potential to *increase* social inequalities in health as it does to *reduce* social inequalities in health. Here, we can revisit our original question, *Who is empowered by these technologies?* It is well known that higher SES individuals tend to adopt innovative technologies earlier than lower SES individuals and often accumulate benefits from early adoption that are unavailable to later adopters [12]. It is also documented that variations in the use of health technologies tend to benefit high SES individuals [9, 11]. These effects are expected to be particularly strong for technologies that are most often accessed and used directly by end-users (such as consumer and home-based technologies), exactly the technologies that increasingly characterize the political priorities expressed in the current discourse [9]. The availability, and informed use, of these resources are particularly dependent on the physical and non-physical resources already at an individual’s disposal, including quality of housing, finances, social network, and health and technology literacy.

It is also expected that high SES individuals would, due to their level of engagement with the technologies, be more influential in development and implementation processes [12]. There is a risk that high SES individuals are often better represented by research and data, market forces, and political power that, in turn, shape the way these technologically innovative resources are developed, adopted and implemented in society [11–13]. Electronic patient journals serve as an interesting example. These technological tools are, in Norway, theoretically available to every citizen (national system). However, physical access is further dependent on stable internet connection and an electronic device that is capable of connecting to the internet [4, 10]. Given physical access, the ability to effectively use these tools, as well as transfer the information in these journals to meaningful benefits for health and care, is dependent on an individual’s cultural, legal, technical and medical literacy level [4, 10]. Furthermore, due to mechanisms referenced earlier, the most active and engaged users of these technologies (i.e. high SES individuals and health care professionals) will inspire development and implementation processes, further solidifying this tool’s usefulness and personalization for already privileged user groups.

The unfortunate result is, again, what can be characterized as a technology-based double burden for low SES individuals, who are less likely to be empowered by these technologies and more likely to be alienated from the potential benefits of these resources over time. It is unclear if the role and responsibility of municipalities represented in the discourse may contribute to a reproduction of these individual inequalities or instead prevent the growth of individual inequalities while reproducing regional inequalities.

## Conclusion

Despite an overt political imperative for reducing social inequalities in health, the Norwegian political discourse surrounding health technologies presented here gives little attention to the potential for these innovations, such as e-Health and welfare technologies, to unintentionally (re) produce social inequalities. Instead, the discourse is characterized by neoliberal undertones that individualize and commercialize public health and promote a pro-innovation ideology. Broader social concerns with implications for unequally (re) distributing resources and power in society are left largely unaddressed.

The potential of health technologies to deliver positive aggregate value to society is dependent on broad recognition of these often unintended and undesired social consequences. These perspectives should be further integrated into policy agendas if the development, adoption and implementation of innovative health technologies is to, in fact, contribute to equal empowerment and a reduction of social inequalities in health. As these technologies increasingly occupy ‘every possible temporal and spatial location’ in society, they contribute to a growing medicalization of society [2, 5]. The increased promotion of these technologies as tools for monitoring and surveillance increase the potential for issues of social control and domestication [2, 7]. These, and related issues, are likely to be unequally shouldered by underprivileged groups in society. Therefore, as it becomes more difficult for individuals to opt-out of the technological imperative, political discourses that uncritically promote these innovations will encourage a form of enforced social coercion and, consequently, an abuse of political power. Therefore, recognizing and addressing these issues requires a critical perspective towards the dominant political discourse to understand how it may systematically undermine, even legally mandated, efforts to reduce social inequalities. Moreover, although one must be careful when generalizing the findings from this analysis to a larger international context, it should be noted that the mechanisms driving the current discourse are neither unique to a Norwegian nor a Scandinavian context and are instead often a product of international sociopolitical and socioeconomic trends. Therefore, we would expect to find similar discourses and similar consequences across national and continental divides, particularly where health technologies form a political and/or economical imperative.

## Figures and Tables

**Fig. 1:**
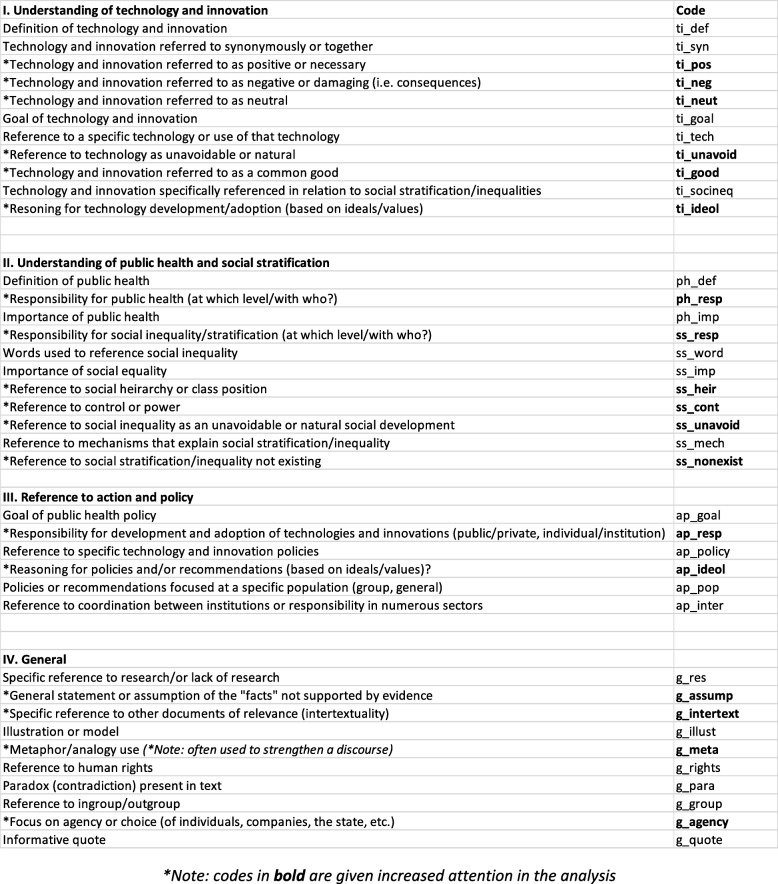
Coding form (in full)

**Fig. 2:**
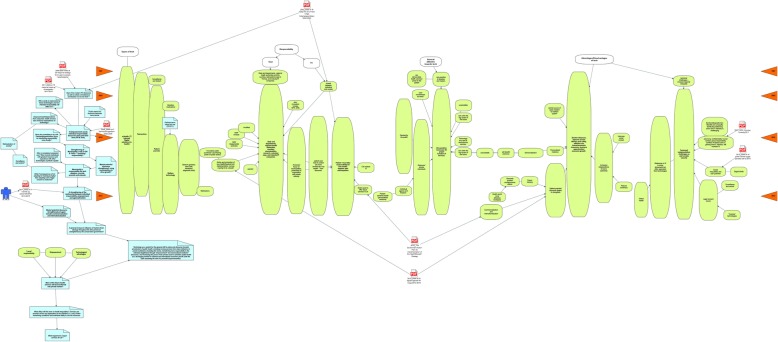
Coding form (in full) used during analysis of the relevant texts

## Data Availability

The datasets used and/or analyzed during the current study are available from the corresponding author on request.

## Appendix 1

**Table 1:** Overview of documents included in the discourse analysis

Document	Document Type	Publishing Organization	Parts of the document analyzed	Year Published
^†^Mer Helse for hver Bit	Action plan	Sosial- og helsedepartementet (Social and Health Ministry)	In full	1997
^††^Telemedisin I Norge	Technical report	Sosial- og helsedepartementet (Social and Health Ministry)	Chp: 1, 3.1, 5.1, 5.3, 5.4, 5.5, 5.6, 6.1, 6.2, 6.3, 6.4, 7.1, 7.2	1998
^†^Si @ Statlig Tiltaksplan	Action plan	Sosial- og helsedepartementet (Social and Health Ministry)	In full	2001
^††^St. meld. 16 Resept for et sunnere Norge	White paper	Helse- og omsorgsdepartementet (Health and Care Ministry)	Chp: 1.1–1.5.2, 5, 10.1.1, 10.3.2	2002
^†^Samspill 2007, Elektronisk samarbeid i helse- og sosialsektoren	Action plan	Sosial- og helsedepartementet (Social and Health Ministry)	In full	2004
^††^St. meld. 20 Nasjonal strategi for å utjevne sosiale helseforskjeller	White paper	Helse- og omsorgsdepartementet (Health and Care Ministry)	Chp: 1.1–1.6, 7.2.2, 7.2.3	2007
^†^Helsevesenet 2013 IKT Perspektivet	Technical report	Kompetansesenter for IT i helse- og sosialsektoren, KITH (Competence center for IT in health & social sector)	In full	2008
^††^St. meld. 7 Et nyskapende og bærekraftig Norge	White paper	Helse- og omsorgsdepartementet (Health and Care Ministry)	Chp: 1.1–1.3.3, 2.1, 2.2, 2.3, 2.4.1, 2.4.2, 8.2–8.2.4	2008
^†^St. meld. 47 Samhandlingsreformen	White paper	Helse- og omsorgsdepartementet (Health and Care Ministry)	Forward; chp: 1, 2, 3.4.1	2008
^†^Samspill 2.0 strategiplan 2008–2013	Action plan	Helse- og omsorgsdepartementet (Health and Care Ministry)	In full	2008
^†^Gode helseregistre - bedre helse 2010–2020	Action plan	Helse- og omsorgsdepartementet (Health and Care Ministry)	Summary; introduction; chp: 5–5.3, 6.4, 8–8.3, 9–9.1, 9.4, 10.2.3, 10.2.10, 11.2, 11.4	2010
^††^NOU innovasjon i Norge	Public inquiry	Helse- og omsorgsdepartementet (Health and Care Ministry)	Chp: 7	2011
^†^St. meld. 16 Nasjonal helse- og omsorgsplan 2011–2015	White paper	Helse- og omsorgsdepartementet (Health and Care Ministry)	Summary; chp: 2, 3, 3.1, 3.3, 4, 5, 6, 6.3, 7, 8, 8.4, 8.5	2011
^††^Nasjonal strategi for bioteknologi 2011–2020	Action plan	Kunnskapsdepartementet (Knowledge Ministry)	Forword; summary; chp: 1, 2.3, 3.5, 4.1	2011
^†^Regjerings digitaliseringsprogram	Action plan	Departementene (The Ministries)	Foreward; summary; chp: 4.2	2012
^†^St. meld. 9 Én innbygger, én journal	White paper	Helse- og omsorgsdepartementet (Health and Care Ministry)	In full	2012
^†^St. meld. 10 Gode kvalitet, trygge tjenester	White paper	Helse- og omsorgsdepartementet (Health and Care Ministry)	Summary; chp: 4–4.8, 6.4, 8.1, 9, 9.3–9.3.2	2012
^†^St. meld. 11 Personvern	White paper	Kongelege fornyings-, administrasjons- og kyrkjedepartementet (Royal Renewal, Administration and Chruch Ministry)	Summary; chp: 2, 2.1, 2.4.4, 2.4.10, 4.2, 4.10, 5.5, 5.6, 5.9, 6.5, 6.6, 7.7, 8.11, 9.1, 9.3.3, 9.7, 10.9, 11	2012
^†^St. meld. 23 Digital agenda for Norge	White paper	Kongelege fornyings-, administrasjons- og kyrkjedepartementet (Royal Renewal, Administration and Chruch Ministry)	Introduction; chp: 2, 3.1.1, 6, 10, 11, 12	2012
^†^St. meld. 29 Morgendagens omsorg	White paper	Helse- og omsorgsdepartementet (Health and Care Ministry)	Introduction; summary; chp: 7, 8, 9	2012
^†^St. meld. 34 Folkehelsemelding	White paper	Helse- og omsorgsdepartementet (Health and Care Ministry)	Introduction; chp: 2.1.2, 3.1, 3.1.1, 3.1.2, 3.1.3, 3.1.4, 6.2.2	2012
^††^Fagrapport om implementering av. velferdsteknologi i de kommunale helse- og omsorgstjenestene 2013–2030	Technical report	Helsedirektoratet (Health Directorate)	Forward; introduction; summary; chp: 1, 2, 3, 6, 7, 8, 9, 10, 11, 12, 13, 14, 15, 16, 17, 18	2012
^††^Helseomsorg21 – Et kunnskapssystem for bedre folkehelse	Action plan	Helse- og omsorgsdepartementet (Health and Care Ministry)	Pages: 6–8, 13–18, 36, 38, 42, 45, 47, 49, 50–52, 54, 61, 79, 80–82, 85–88, 89, 91, 100–110, 113, 115, 117, 118, 121, 125–130, 133, 134, 140, 143, 144, 146, 149, 153, 155	2014
^†^St. meld. 11 Kvalitet og pasientsikkerhet	White paper	Helse- og omsorgsdepartementet (Health and Care Ministry)	Chp: 1, 9 9.1, 9.2, 9.3, 9.4	2014
^†^St. meld. 19 Folkehelsemelding	White paper	Helse- og omsorgsdepartementet (Health and Care Ministry)	Chp: 1–1.5, 3.1, 4.5, box 4.7, 9.1.1, 9.1.2	2014
^†^St. meld. 26 Fremtidens primærhelsetjeneste	White paper	Helse- og omsorgsdepartementet (Health and Care Ministry)	Chp: 1–5, 15.4, 18–18.5	2014
^†^St. meld. 28 Leggemiddelmeldingen	White paper	Helse- og omsorgsdepartementet (Health and Care Ministry)	Chp: 1, 2–2.4, 3–3.4, 4–4.7, 7.1.3, 7.2.1, 8, 18, 19, 19.4.4, 20, 21, 22.4, 22.4.3, 22.5.3, 23–23.4, 24–24.1.3	2014
^††^Første gevinstrealiseringsrapport – Nasjonalt velferdsteknologiprogram	Technical report	Helsedirektoratet (Health Directorate)	Chp: 1.1, 1.2, 2, 3, 5.1, 5.2, 5.3	2015
^†^St. meld. 11 Nasjonal helse- og sykehusplan	White paper	Helse- og omsorgsdepartementet (Health and Care Ministry)	Chp: 1,2,3, 4, 4.1, 4.2, 5, 5.1, 5.3, 5.4, 6, 7, 7.5, 9, 9.7, 12.4, 15, 15.1, 15.2, 19, 21.3	2015
^††^The Government action plan for implementation of the Health&Care21 strategy	Action plan	Departementene (The Ministries)	Foreword; introduction; chp: 8, 10, 13, 14, 15, 23, 24, 27, 28	2015
^††^Andre gevinstrealiseringsrapport – Nasjonalt velferdsteknologiprogram	Technical report	Helsedirektoratet (Health Directorate)	Chp: 1.1, 1.2, 2.1, 2.2, 4.1–4.3	2017
^†^Nasjonal e-helse strategi og mål 2017–2022	Action plan	Direktoratet for e-Helse (Directorate for e-Health)	In full	2017
^†^Nasjonal handlingsplan for e-helse 2017–2022	Action plan	Direktoratet for e-Helse (Directorate for e-Health)	In full	2017

^†^Documents included by recommendation from official Norwegian directorates

^††^Documents included from hand search or snowball method

## Abbreviations

CDA: Critical discourse analysis
EPJ’s: Electronic patient journals
ICT: Information and communication technologies
KITH: Norwegian Competence center for information technologies in the health and social sector
SES: Socioeconomic status
